# Evaluating the Therapeutic Effect of Hispidin on Prostate Cancer Cells

**DOI:** 10.3390/ijms25147857

**Published:** 2024-07-18

**Authors:** Kai-Cheng Chan, Praveenkumar Basavaraj, Jui-Chen Tsai, Jonathan Viehoever, Bing-Yan Hsieh, Xin-Yu Li, Guan-Jhong Huang, Wen-Chin Huang

**Affiliations:** 1 Graduate Institute of Cell Biology, College of Life Sciences, China Medical University, Taichung 40402, Taiwan; u109210005@cmu.edu.tw (K.-C.C.); praveenb@mail.cmu.edu.tw (P.B.); 2 Department of Medical Laboratory Science and Biotechnology, China Medical University, Taichung 40402, Taiwan; u110007410@cmu.edu.tw (J.-C.T.); u110007315@cmu.edu.tw (B.-Y.H.); u110007302@cmu.edu.tw (X.-Y.L.); 3 International Master’s Program of Biomedical, School of Medicine, China Medical University, Taichung 40402, Taiwan; u111206142@cmu.edu.tw; 4 Department of Chinese Pharmaceutical Sciences and Chinese Medicine Resources, College of Chinese Medicine, China Medical University, Taichung 40402, Taiwan; gjhuang@mail.cmu.edu.tw; 5 Department of Food Nutrition and Healthy Biotechnology, Asia University, Taichung 41354, Taiwan

**Keywords:** anti-prostate cancer therapy, apoptosis, hispidin, MMP, nature compound

## Abstract

Androgen deprivation therapy (ADT) is the primary treatment for advanced prostate cancer (PCa). However, prolonged ADT inevitably results in therapy resistance with the emergence of the castration-resistant PCa phenotype (CRPC). Hence, there is an urgent need to explore new treatment options capable of delaying PCa progression. Hispidin (HPD) is a natural polyketide primarily derived from plants and fungi. HPD has been shown to have a diverse pharmacological profile, exhibiting anti-inflammatory, antiviral, cardiovascular and neuro-protective activities. However, there is currently no research regarding its properties in the context of PCa treatment. This research article seeks to evaluate the anti-cancer effect of HPD and determine the underlying molecular basis in both androgen-sensitive PCa and CRPC cells. Cell growth, migration, and invasion assays were performed via the MTS method, a wound healing assay and the transwell method. To investigate if HPD affected the expression of proteins, Western blot analysis was conducted. Furthermore, apoptosis was assessed by Annexin V-FITC/PI staining and Western blot analyses. HPD exhibited a favorable pharmaceutical profile to inhibit cell growth; disrupt the cell cycle; attenuate wound healing, migration and invasion; and induce apoptosis in PCa cells in vitro. The mechanistic results demonstrated that HPD reduced AR, MMP-2 and MMP-9 expression and activated the caspase-related pathway, leading to programmed cell death in PCa cells. We showed the anti-cancer effect of HPD on PCa cells and confirmed its feasibility as a novel therapeutic agent. This study provides significant insights into the delineation of the molecular mechanism of HPD in PCa cells and the development of an effective and safe therapy using HPD to eliminate PCa progression.

## 1. Introduction

The prostate is a vital component of the male reproductive system located within the pelvic cavity, positioned below the bladder and anterior to the rectum. The main function of the prostate lies in the production and reservoir of prostatic fluid [1]. However, prostate cancer (PCa) is the unstrained growth of cells in the prostate, with the highest incidence rate of new cases being found in the USA and Western countries [2,3]. PCa could be propelled by the global adoption of Westernized diets, an aging demographic and refined diagnostic technologies. Due to its often slow progression, the early detection of PCa poses a formidable challenge. As such, the diagnosis of this malignancy mostly occurs only subsequent to metastasis to critical sites, including bone, which significantly heightens the complexity of therapeutic intervention [4]. Common treatment strategies include radical prostatectomy [5] and radiation therapy [6] for local PCa, systemic androgen deprivation therapy (ADT) [7] for advanced PCa and chemotherapy [8] for terminal PCa. While pharmacological ADT is effective for managing symptoms and slowing the progression of advanced PCa, it is associated with severe adverse effects [9] and inevitably leads to the development of the castration-resistant phenotype (CRPC), typically within a five-year frame [10]. Therefore, there is a critical need for the exploration of new and promising agents that promise to hinder the transition of PCa cells into CRPC or directly kill PCa cells, coupled with an elucidation of their molecular regulatory mechanisms, for future application in clinical treatment.

Hispidin (HPD) is a naturally occurring polyketide product ubiquitously found in several plants and fungi [9,10,11,12], and it exhibits a wide array of pharmacological activities. Previously, the antioxidant [13], anti-inflammatory [14], antiviral [15] and cardiovascular- [16] and neuro-protective [17] capabilities of HPD have been evaluated. Polyketide compounds, but not HPD, have been reported to display cellular toxic activity in PCa cells [18]. Additionally, studies of murine models have affirmed the biological safety profile of HPD, with no adverse effects [9,19]. Intriguingly, HPD has also demonstrated a substantial inhibitory effect on growth and survival in gastrointestinal cancer models, including stomach [20], pancreatic [21] and colorectal cancer [22]. However, the specific anti-cancer function and the underlying molecular mechanism of HPD in PCa remain to be uncovered.

In the present study, we evaluated the anti-cancer effect and determined the molecular basis of HPD in PCa cells. HPD affected cell cycle progression at the S phase, resulting in a substantial inhibition of cell growth in both LNCaP (androgen-sensitive) and C4-2 (CRPC) PCa cells. Furthermore, HPD significantly suppressed migration and invasion by interference with the protein expression of matrix metalloproteinase (MMP)-2 and MMP-9. Moreover, HPD induced PCa cell death through activation of caspase-associated apoptosis. As such, this study demonstrates, for the first time, that HPD exhibits emerging anti-cancer efficacy in PCa. These findings offer a new insight into the molecular mechanism of HPD in PCa cells and provides the basis for potential future application of HPD in the management of PCa progression.

## 2. Results

### 2.1. HPD Attenuated the Growth of PCa Cells

**Figure 1:**
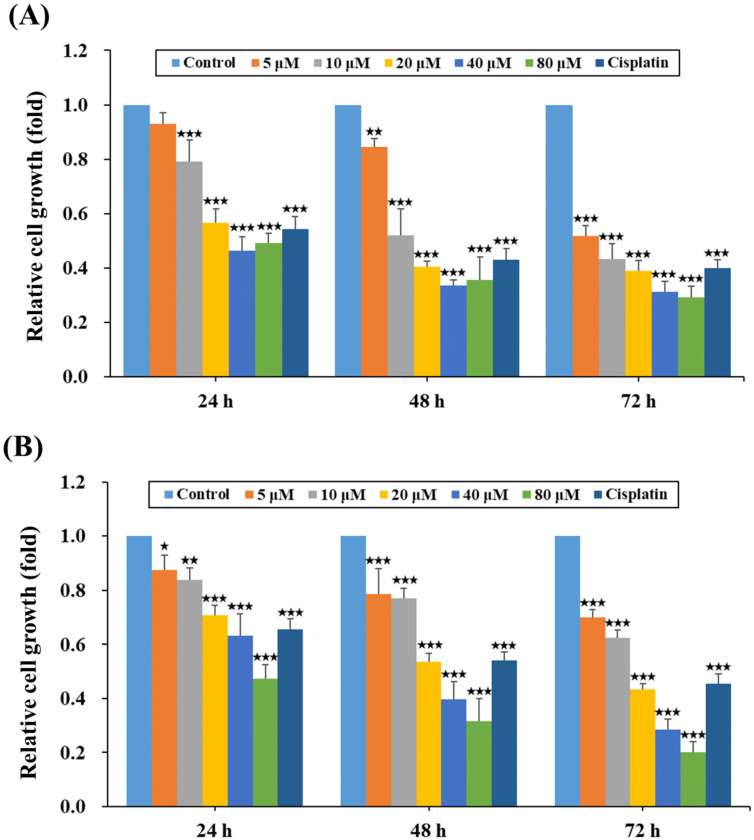
HPD inhibited cell growth in PCa cells. (**A**) LNCaP and (**B**) C4-2 cells were treated with control or HPD for 24, 48 and 72 h. The relative cell growth was assigned as the fold of the control-treated cells for 24, 48 and 72 h, respectively. The relative fold was defined as the ratio of each dose of HPD to the control group (as 1.0). Results were shown as the mean ± SD of three independent experiments. Cisplatin (10 µM) was employed as a positive control. * *p* < 0.05, ** *p* < 0.01, *** *p* < 0.001.

HPD is a natural polyketide and has been reported to display multiple favorable pharmacological functions with low toxicity in normal cells [9,19]. To evaluate the therapeutic impact of HPD on the cellular properties of PCa, several bio-functionality assays were performed, including cell growth, migration and invasion, as well as cell death analysis. Firstly, LNCaP and C4-2 PCa cells were subjected to dose-dependent HPD treatment to study its effects on cell growth, utilizing an MTS proliferation assay at 24-, 48- and 72-h time points. The results revealed a profound impact of HPD on the growth rates of LNCaP (Figure 1A) and C4-2 (Figure 1B) cells across different concentrations in a dose-dependent manner, with an achieved IC_50_ (half-maximal inhibitory concentration) of 6.09 μM for LNCaP cells and 16.63 μM for C4-2 cells, respectively, at 72 h of treatment. Moreover, we considered that hormones (such as androgens) in fetal bovine serum (FBS) might affect the efficacy of HPD on androgen-sensitive LNCaP PCa cells. An additional experiment was included using charcoal-stripped FBS, which had been stripped of hormones and non-polar materials (lipophilic compounds). As shown in Supplemental Figure S1, HPD presented the similar effects on LNCaP cell growth in regular FBS and in charcoal-stripped FBS. It suggested that HPD would not change the efficacy of cell growth in androgen-sensitive PCa cells with or without the androgen condition.

### 2.2. HPD Impeded Cell Cycle Progression at the S Phase in PCa Cells

**Figure 2:**
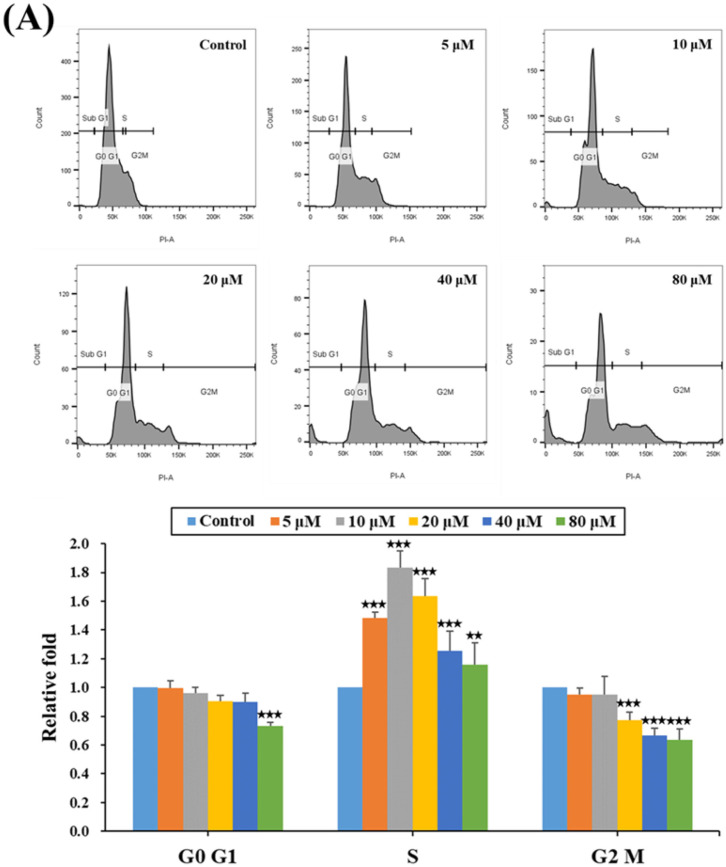

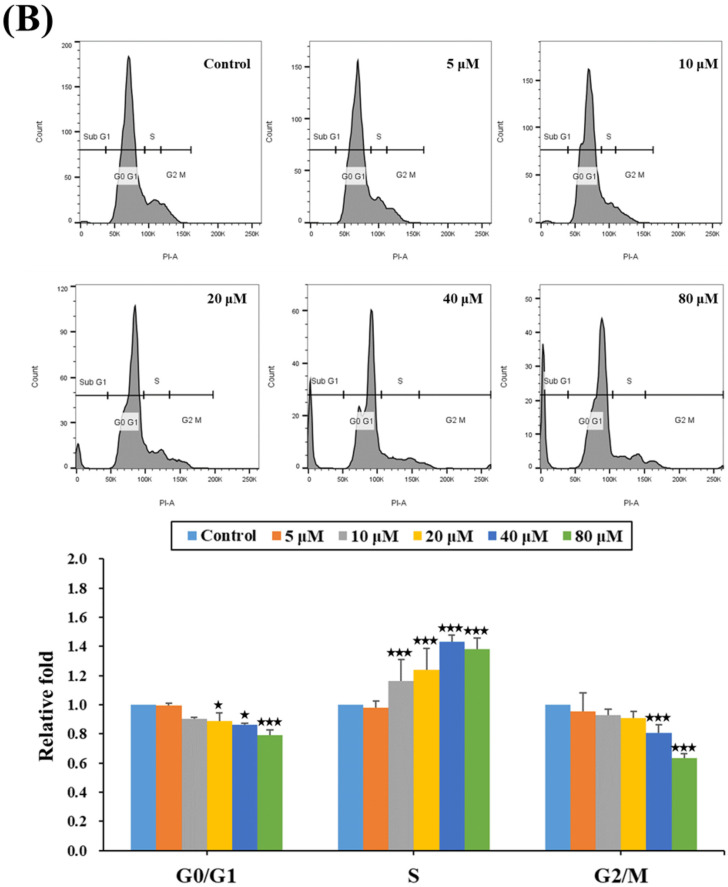
HPD induced cell cycle arrest, as indicated through increases in the S-phase PCa cell numbers. (**A**) LNCaP and (**B**) C4-2 cells were treated with HPD and control for 24 h, followed by flow cytometry. The relative fold was assigned relative to the control-treated cells for each phase. The representative images of flow cell cycle are displayed in the top panels and the quantification of the results are in the bottom panels. The relative fold was defined as the ratio of each dose of HPD to the control group (as 1.0). Results are represented as the mean ± SD of three independent experiments. * *p* < 0.05, ** *p* < 0.01, *** *p* < 0.001.

Given the importance of the cell cycle for cell growth, we conducted a thorough examination of the cell cycle phase distribution by employing flow cytometry analysis after treating PCa cells with HPD at various concentrations. As shown in Figure 2A (LNCaP) and Figure 2B (C4-2), a significant dose-dependent increase in the relative fold of cell populations at the S phase was observed after HPD treatment. Concurrently, the relative fold of cell populations at the G2/M phase was reduced. Notably, an additional cell population in the sub-G1 phase was detected at high concentrations of HPD (Supplemental Figure S2), indicating possible cell death induction by HPD. Collectively, the data suggested that HPD affected cell cycle progression at the S phase and induced a sub-G1 cell population in PCa cells, resulting in the substantial inhibition of PCa cell growth and induction of cell death.

### 2.3. HPD Suppressed Migration and Invasion of PCa Cells

**Figure 3:**
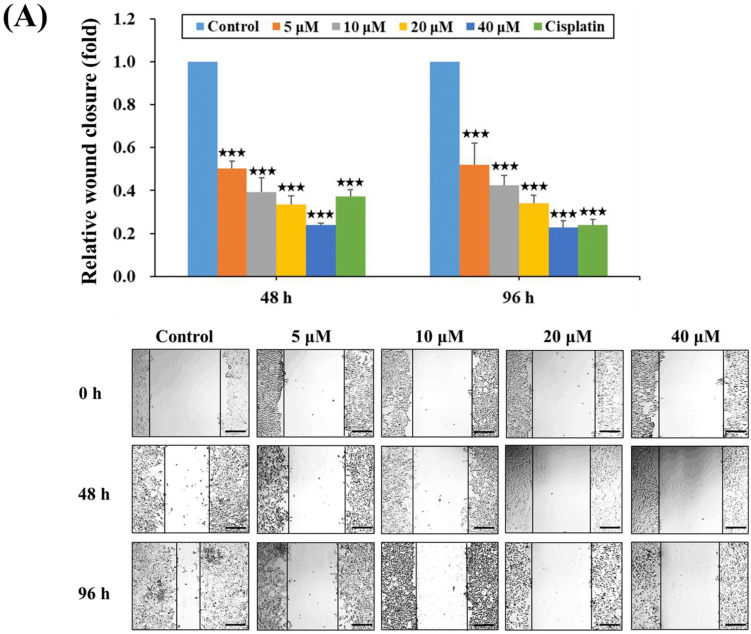

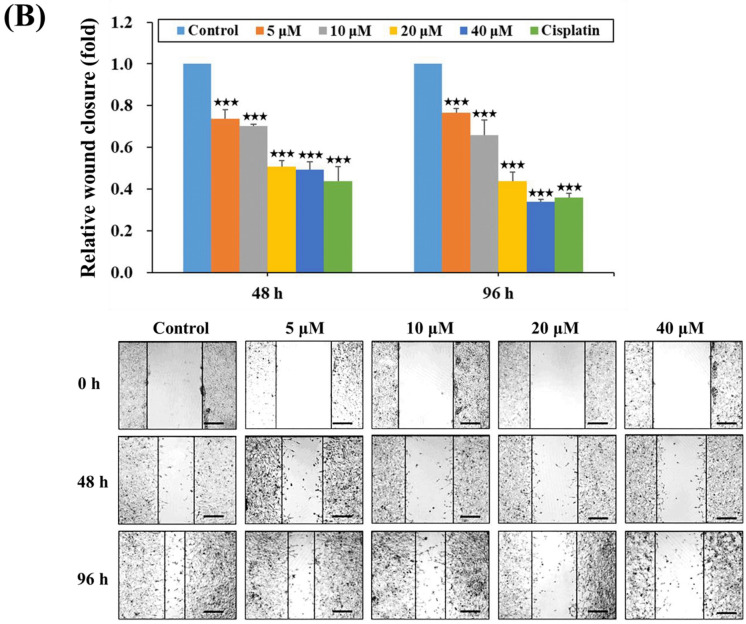
HPD inhibited the migratory ability of PCa cells. (**A**) LNCaP and (**B**) C4-2 cells were treated with control or HPD for 48 and 96 h. The migratory distances of PCa cells at each concentration of HPD or the control were compared to the baseline at 0 h. The relative cell migratory distances were calculated to assess the wound healing capabilities of cells at 48 and 96 h (control as 1.0). The quantification of wound closure (the top panels) and the representative images of wound closure (the bottom panels; Scale bar = 100 µm) are shown. Results represented as the mean ± SD of three independent experiments. Cisplatin (10 µM) was used as a positive control. *** *p* < 0.001.

We subsequently employed a wound healing assay to determine the influence of HPD on the migratory ability of PCa cells. Comparing the migratory patterns of HPD-treated LNCaP (Figure 3A) and C4-2 (Figure 3B) cells, the results revealed significant reductions in the relative migratory distances compared to their respective controls. Thus, HPD possessed the capability to affect migration in PCa cells in a concentration-dependent manner.

**Figure 4:**
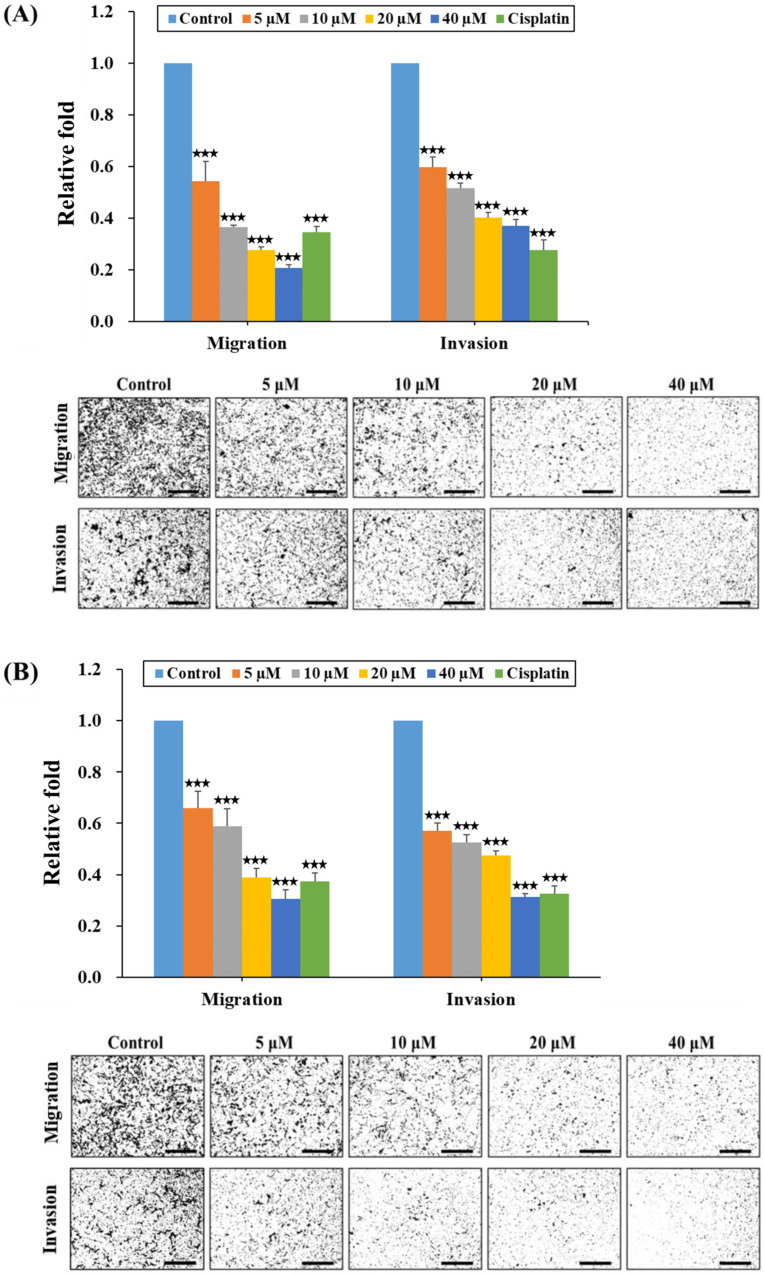
HPD inhibited the migratory and invasive potentials of PCa cells. The transwell migration and invasion assays were performed to determine migration and invasion in both (**A**) LNCaP and (**B**) C4-2 cells in accordance with the control and HPD treatment. The relative migration and invasion were assigned as 1.0 in the control-treated cells. The relative cell numbers for migration and invasion were calculated (control as 1.0). The quantification of results (the top panels) and the representative images of transwell migration and invasion (the bottom panels; Scale bar = 100 µm) were shown. Results are represented as the mean ± SD of three independent experiments. Cisplatin (10 µM) was applied as a positive control. *** *p* < 0.001.

Next, we assessed the effects of HPD on the migration and invasion dynamics of PCa cells by employing the transwell method. Equal numbers of LNCaP and C4-2 cells were seeded into the transwell chambers, being subjected to either the control condition or various concentrations of HPD treatment for 48 h. As shown in Figure 4A (LNCaP) and Figure 4B (C4-2), HPD significantly reduced both the migratory and invasive capabilities of PCa cells, aligning with the observations shown in Figure 3A,B. Remarkably, the inhibitory effects of HPD commenced at a concentration as low as 5 μM. The combined data indicated that HPD greatly suppressed the migration and invasion of LNCaP and C4-2 cells.

### 2.4. HPD Reduced Protein Expression Related to Cell Growth and Cancer Progression in PCa Cells

**Figure 5:**
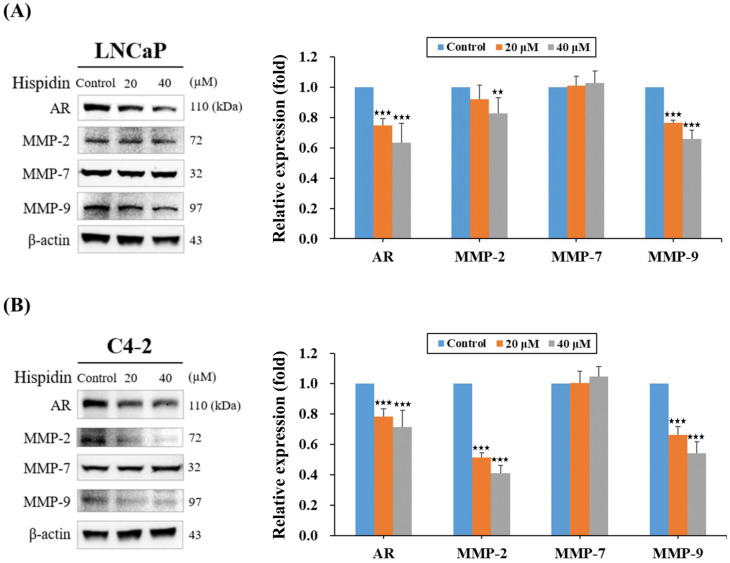
HPD reduced the expression of AR, MMP-2 and MMP-9 proteins in PCa cells. (**A**) LNCaP and (**B**) C4-2 cells were treated with HPD or control for 48 h. The expression levels of AR and MMP proteins were subsequently detected by Western blot analysis. The relative protein expression (fold) was assigned as 1.0 in the control-treated cells. The results of Western blot analysis (the left panels) and the quantitative assays of protein expression (the right panels) were shown. Results are represented as the mean ± SD of three independent experiments. β-actin was used an internal control. ** *p* < 0.01, *** *p* < 0.001.

AR and MMPs have been well established as essential protein factors associated with cell growth, cell cycle regulation, and migration and invasion in PCa [23,24,25,26]. To delineate whether HPD affected these key proteins, Western blot analysis was conducted. The results revealed that HPD had inhibitory effects on AR expression in a dose-dependent manner in both LNCaP and C4-2 cells (Figure 5A,B). Additionally, concentration-dependent reductions in the expression levels of MMP-2 and MMP-9 proteins in PCa cells after HPD treatment was observed (Figure 5A,B). However, no significant change in the expression levels of MMP-7 was detected in both cells. Taken together, the results of Western blot analysis suggested that HPD reduced the expression levels of AR, MMP-2 and MMP-9 proteins, all linked to cell progression leading to the inhibition of growth (Figure 1), migration and invasion (Figure 3 and Figure 4) in PCa cells.

### 2.5. HPD Induced Cell Death via Activation of Caspase-Dependent Apoptosis in PCa Cells

**Figure 6:**
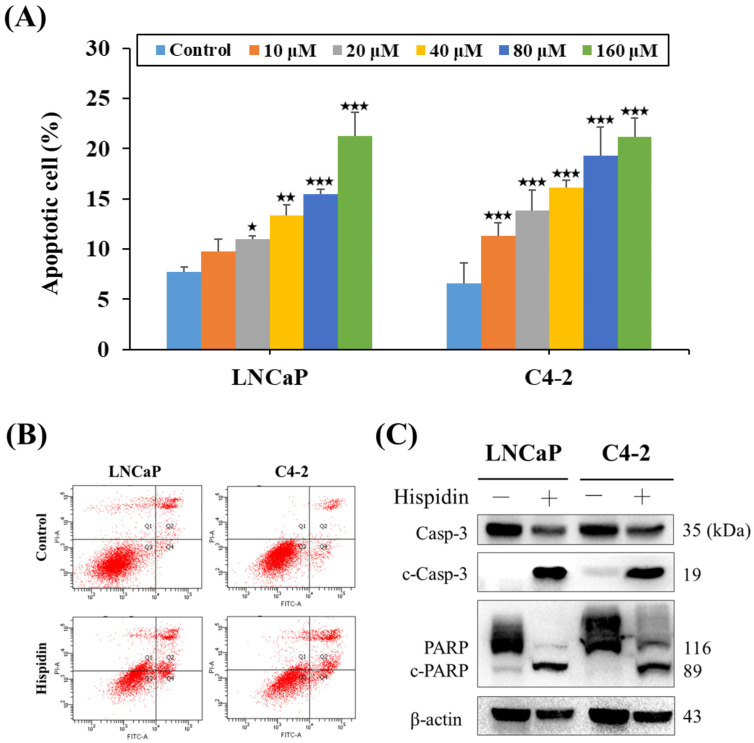
HPD induced caspase-dependent cell death in PCa cells. (**A**) PCa cells were exposed to control or HPD for 48 h, and apoptotic cells were subsequently analyzed by a flow cytometry-based Annexin V/PI dual-staining assay. Results represented as the mean ± SD of five independent experiments. (**B**) Representative red dot plots of PCa cells treated with control or HPD (160 μM) from (**A**). (**C**) Western blot analysis of caspase-3 (Casp-3), cleaved caspase-3 (c-Casp-3), PARP and cleaved PARP (c-PARP) expression in PCa cells after HPD or control treatment. β-actin was used as an internal control. * *p* < 0.05, ** *p* < 0.01, *** *p* < 0.001.

Previously, we demonstrated that HPD induced the sub-G1-phase cell population linked to cell death in PCa cells. To further unravel the underlying molecular basis re-sponsible for HPD-mediated cell death, we first utilized flow cytometry with Annexin V and PI staining. As shown in Figure 6A, the dual-staining results showed that the percentages of apoptotic LNCaP and C4-2 cells were as low as 7.7 ± 0.5% and 6.6 ± 1.4% in the control groups, respectively. Significantly, HPD raised the apoptotic cell fractions in a dose-dependent manner (Figure 6A,B). Subsequently, the expression levels of caspase-3 as an essential executioner caspase for apoptosis and its downstream factor, poly (ADP-ribose) polymerase (PARP), were analyzed by Western blotting to evaluate if caspase-dependent apoptosis was activated by HPD treatment. As shown in Figure 6C, in comparison with the control group, HPD decreased the expression levels of caspase-3 (Casp-3; 35 kDa) and PARP (116 kDa) proteins. However, the activated forms of Casp-3 and PARP, cleaved Casp-3 (c-Casp-3; 19 kDa) and cleaved PARP (c-PARP; 89 kDa), were substantially increased after HPD treatment (Figure 6C). These findings suggested that HPD was able to induce cell death via activation of caspase-dependent apoptosis in PCa cells, culminating in the cleavage of Casp-3 into c-Casp-3 and subsequent cleavage of downstream PARP into c-PARP, thereby fostering the breakdown of PCa cells and inducing apoptosis.

## 3. Discussion

PCa progression, including CRPC, poses a challenging and unresolved clinical issue in PCa research. Currently, there is no effective remedy available to successfully cure this deadly disease. Crude extracts and/or pure compounds directly isolated from herbal plants and fungi belonging to the *Phellinus* and *Inonotus* genera have been demonstrated to be effective at treating diseases, including cardiovascular and hepatic diseases, as well as diabetes [27,28]. Specifically, HPD is purified from *Phellinus* and *Inonotus* and displays pharmacological effects with antioxidant [13], anti-inflammatory [14] and cardiovascular- [16] and neuro-protective [17] activities. In this study, we elucidated the efficacy of HPD in the treatment of PCa in vitro for the first time and provided the underlying molecular basis in both androgen-sensitive LNCaP and CRPC C4-2 cells. Interestingly, a very recent paper reported that HPD also substantially influenced metastatic PCa cells though alternations of the PI3K and the MAPK signaling pathways, as well as changes in the status of mitochondrial reactive oxygen species (ROS) in PC-3 and DU145 cells [29]. These findings particularly strengthened our study and enriched the potential and multiple biological activities of HPD in PCa. But, few possible limitations associated with the use of HPD have recently been identified, such as the fact that the chemical instability of HPD easily lost its antioxidant activity because it is susceptible to air oxidation [30]. Additionally, HPD showed lower cell permeability for stage 2 human colorectal carcinoma and blood–brain barrier penetration [31]. It needs to further investigate the potential challenges of HPD pharmacokinetics for the clinical application. 

One prominent cancer hallmark is uncontrolled cell growth and division [32] linked to the cell cycle. Specific blockade of the cell cycle elements could be considered and employed as a possible therapeutic strategy in the treatment of cancer [33,34,35]. The cell cycle is restrictively regulated to ensure that cells undergo DNA replication and division at appropriate times, typically including the G0, G1, S, G2, and M phases. The G0 phase refers to the period when cells remain dormant; they are metabolically active, but not proliferating. After re-entering the cell cycle, in the G1 phase, cells begin to proliferate, marking the period of highest DNA content as they prepare for entry into the S phase. During the S phase, DNA replication occurs, resulting in a doubling of the diploid content. Afterwards, in the G2 phase and the M phase, cells enter the division process into two daughter cells. Our data revealed that HPD significantly raised the percentage of PCa cells in the S phase of the cell cycle (Figure 2). Concurrently, there was a decrease in the percentage of PCa cells in the G2/M phases. These results demonstrated that the HPD-treated PCa cells accumulated in the S phase, impeding progression towards the G2/M phase, thus disrupting the completion of the cell cycle to inhibit PCa cell growth.

Advanced PCa often forms metastasis [4], making the inhibition of their migration and invasion crucial in PCa treatment. In particular, our results demonstrated that HPD significantly suppressed migration and invasion in LNCaP and C4-2 cells as low as 5 μM in a dose-dependent pattern (Figure 3 and Figure 4). MMPs have been demonstrated to play a pivotal role in mediating the synthesis, degradation, and remodeling of the extracellular matrix (ECM) [36,37]. The ECM contains several protein factors, including collagen, fibronectin, gelatin, and other structural proteins, which are fundamental for the maintenance of cellular morphology, movement and intercellular communication, as well as the functions of tumor microenvironment [38]. Importantly, MMPs can degrade and disrupt adhesion between cells and the ECM, promoting the migration and invasion of tumor cells into other tissues and further facilitating the development of fatal metastasis. Among MMPs, MMP-2, -7 and -9 have been reported to promote cancer cell migration and invasion, with MMP-2 and MMP-9 being especially prevalent in PCa progression [39,40,41,42,43]. Thus, targeting these cancer-associated proteases offers an intriguing opportunity to prevent PCa progression under the alternation of the tumor microenvironment. Notably, our data confirmed that HPD significantly reduced the protein expression of MMP-2 and MMP-9 (Figure 5), demonstrating its potential to attenuate the migratory and the invasive capabilities of PCa cells (Figure 3 and Figure 4). Further research is needed to elucidate the specific inhibitory mechanisms and the related signaling pathways.

The activation of programmed cell death/apoptosis has been found to be employed as an attractive therapeutic strategy to treat cancer [44,45,46]. Caspases are a class of cysteine proteases controlling the cellular process of programmed death. This family intricately regulates apoptosis through precise interactions with numerous protein factors [47,48]. Under normal conditions, Casp-3 remains inactive in its original form within the cellular matrix. When cells receive apoptotic stimuli, such as drug treatment, Casp-3 undergoes cleavage, transforming into c-Casp-3, an activated state responsible for cellular apoptosis. Subsequently, PARP is cleaved. Research indicated that c-Casp-3 can cleave PARP (116 kDa), producing c-PARP (89 kDa) as a catalyst for cell disintegration, ultimately leading to cellular apoptosis [49]. Our Western blot data revealed that the expression levels of precursor Casp-3 and PARP proteins were decreased through HPD treatment in LNCaP and C4-2 cells, while the levels of activated c-Casp-3 and c-PARP proteins were increased (Figure 6C). Moreover, flow cytometry analysis confirmed that HPD effectively increased the numbers of apoptotic cells in PCa cells (Figure 6A,B). Taken together, the apoptotic data implied that HPD could serve as a potential therapeutic agent for treating malignant PCa. An additional pre-clinical study will be warranted to evaluate the anti-PCa/CRPC effect of HPD in vivo.

## 4. Materials and Methods

### 4.1. Cell Lines and Cell Culture

PCa cell lines and LNCaP (androgen-sensitive) and C4-2 (CRPC) cells were kindly offered by Dr. Leland W.K. Chung (Cedars-Sinai Medical Center, Los Angeles, CA, USA). PCa cells were cultured and maintained in RPMI 1640 medium (Thermo Fisher Scientific/GIBCO, Waltham, MA, USA) with 10% FBS (GE Healthcare/Hyclone, Pittsburgh, PA, USA) in a humidified 37 °C incubator (Thermo Fisher Scientific) under 5% CO_2_ atmosphere.

### 4.2. Cell Growth Assay

Cell growth was examined by the CellTiter 96^®^ AQ_ueous_ One Solution Cell Proliferation assay (Promega, Madison, WI, USA) [44,50]. Both LNCaP and C4-2 cells were seeded in 96-well plates (1 × 10^4^ cells/well) and incubated overnight. Subsequently, cells were treated with increasing concentrations of HPD (5, 10, 20, 40 and 80 μM) or control (0.08% DMSO) for 24, 48 and 72 h. The HPD (Supplemental Figure S3) was provided by Dr. Guan-Jhong Huang (China Medical University, Taiwan) [51]. Originally, HPD was dissolved in DMSO (a stock solution; 100 mM) and then diluted by RPMI 1640 medium in different working concentrations. Moreover, a common anti-cancer drug, cisplatin (Sigma-Aldrich, St. Louis, MO, USA), was utilized as a positive control [44]. 

### 4.3. Cell Migration Assay

The migratory ability of PCa cells treated with HPD (5, 10, 20 and 40 μM) or control (0.08% DMSO) was evaluated by a wound healing assay [45,46]. Additionally, cisplatin (10 µM) as utilized as a positive control. An artificial “wound” was created using a 200 μL pipette tip on the confluent cell monolayers of the 6-well plates. Cells were recorded under a microscope equipped with a camera at different time points. Quantitative analysis of the wound closure was calculated by the distance traveled by cells using the ImageJ 1.54g software.

### 4.4. Cell Progression Assays In Vitro

Cell progression assays in vitro were conducted by transwell chambers [45,46]. The membranes of the transwell chamber were either used natively (for cell migration assay) or coated with growth factor-reduced Matrigel basement membrane matrix (Coring, Bedford, MA, USA) (for cell invasion assay). Subsequently, PCa cells (1 × 10^5^ cells/well) were seeded in the transwell chamber and then treated with HPD (5, 10, 20 and 40 μM) or the control (0.08% DMSO). Additionally, cisplatin (10 µM) was utilized as a positive control. After 48 h, the migrated or invaded cells were fixed with 4% formaldehyde and stained with a crystal violet solution. After dehydration, an inverted microscope was used to count the numbers of PCa migrated and invaded cells.

### 4.5. Investigation of Cell Cycle Arrest

To understand the HPD-mediated effect on the cell cycle, the phase distribution in PCa cells was detected by flow cytometry. PCa cells were incubated with different concentrations of HPD (5, 10, 20, 40 and 80 μM) or control (0.08% DMSO) for 24 h. Subsequently, the treated PCa cells were centrifuged and cell pellets were fixed with chilled ethanol through 24 h incubation at −20 °C. Eventually, the cells were stained with 20 μg/mL of Propidium Iodide (PI) solution (BioLegend, San Diego, CA, USA) at 37 °C for 30 min and analyzed using FACSCanto (BD Biosciences, San Diego, CA, USA).

### 4.6. Apoptosis Analysis

After being treated for 48 h with HPD or the control, PCa cells were assayed by an Annexin V-FITC and PI Detection Kit (Biolegend, San Diego, CA, USA) based on the manufacturer’s instructions. The percentage of apoptotic PCa cells were detected by a flow cytometer with FCS Express v2.0 software (BD FACSCanto, BD Bioscience, San Diego, CA, USA).

### 4.7. Western Blot Analysis

To examine the protein expression levels, Western blot analysis was performed. Total protein preparation, SDS-PAGE electrophoresis, blotting and protein signal detection were described previously [52]. Additionally, the primary antibodies used in this study were as follows: anti-AR (Santa Cruz Biotechnology, Dallas, TX, USA), MMP-2, MMP-7, MMP-9 (Cell Signaling Technology, Danvers, MA, USA), caspase-3 (Novus Biologicals, Centennial, CO, USA), cleaved caspase-3 (Biovision, Waltham, MA, USA), PARP and β-actin (GeneTex, Irvin, CA, USA).

### 4.8. Statistical Analysis

All data underwent analysis based on a minimum of at least three independent experiments utilizing a two-tailed unpaired Student’s *t*-test to compare independent means. Statistically significant results were defined as *p*-values less than 0.05: * *p* < 0.05, ** *p* < 0.01, and *** *p* < 0.001.

## 5. Conclusions

**Figure 7:**
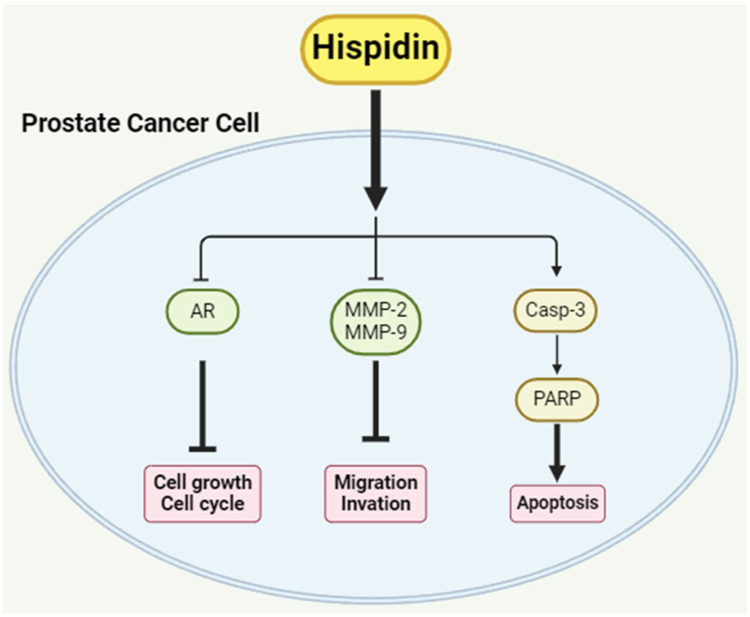
HPD displays the promising therapeutic effect on PCa cells. HPD inhibited cell growth, migration and invasion while simultaneously reducing AR, MMP-2 and MMP-9 expression and activating the Casp-3 associated pathway leading to apoptosis.

Our research data provided evidence that HPD exhibited a favorable pharmaceutical profile to inhibit cell growth, disrupt the cell cycle, attenuate migration and invasion and induce apoptosis in PCa cells (Figure 7). Through the inhibition of AR expression, HPD suppressed PCa cell growth and influenced cell cycle. By decreasing MMP-2 and MMP-9 expression, HPD attenuated migration and invasion in PCa cells. Furthermore, HPD led to apoptosis through the activation of the Casp-3 and PARP pathway in PCa cells. We showed the anti-cancer effect of HPD on PCa cells in vitro and confirmed its feasibility as a novel therapeutic agent. Additionally, a promising safety profile of HPD has been previously reported in the murine models with the low adverse effects [9,19]. Ultimately, we aim to develop an effective and safe therapy using HPD to eliminate PCa and prevent the progression to CRPC.

## Data Availability

Data were contained within the article.

## Supplementary Materials

The following supporting information can be downloaded at: https://www.mdpi.com/article/10.3390/ijms25147857/s1.
