# Supplementary material for: Evaluating the Therapeutic Effect of Hispidin on Prostate Cancer Cells
Source: Int J Mol Sci. 2024 Jul 18;25(14):7857. doi: 10.3390/ijms25147857 (PMC11277327; doi:10.3390/ijms25147857)
Supplement: Supplementary file 1 [file ijms-25-07857-s001.zip › ijms-3045995-supplementary.pdf]

Supplemental Figures

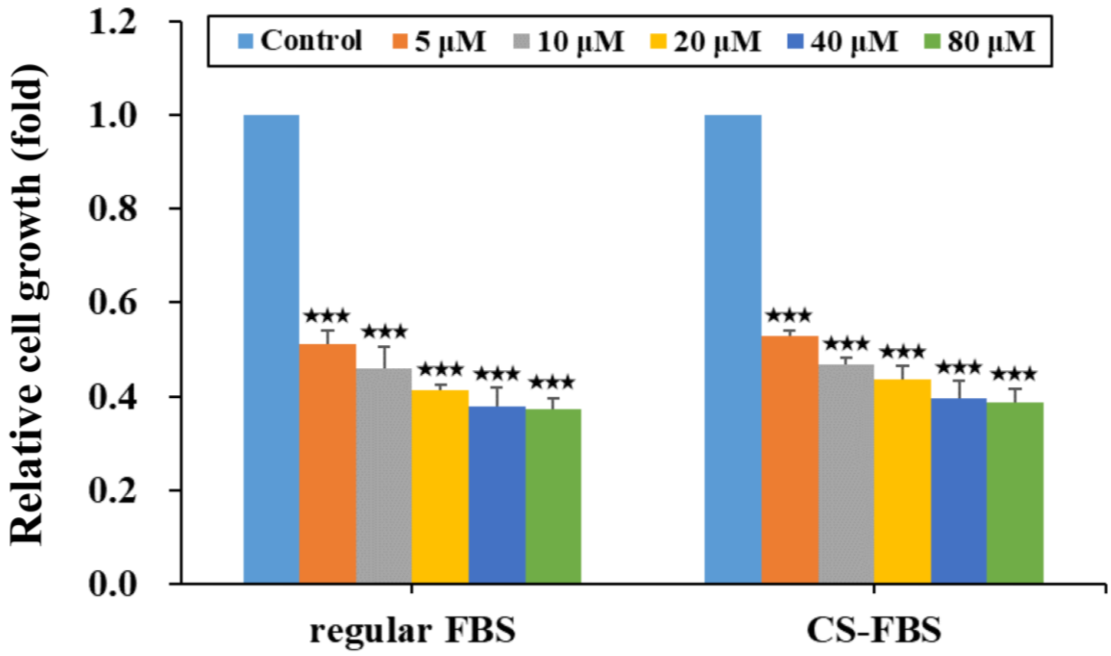

Figure S1. HPD inhibited growth of androgen-sensitive LNCaP cells in the regular FBS and the charcoal-stripped (CS) FBS conditions (72 h). *** $p < 0.001$. Note: IC_{50} of HPD in LNCaP cells were 6.09 µM (regular FBS) and 7.34 µM (CS-FBS), respectively (at 72 h)

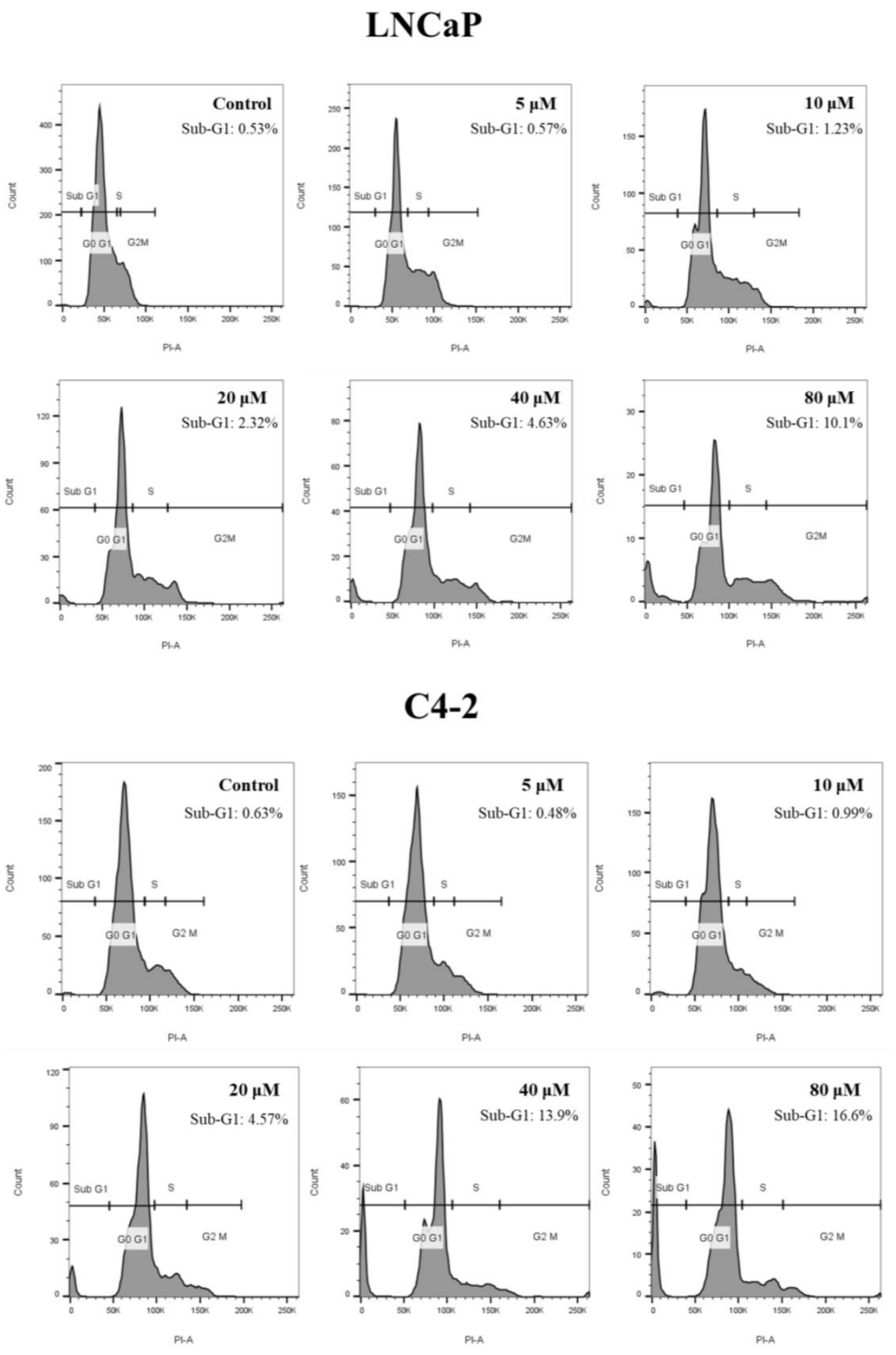

Figure S2. HPD affected the cell populations in the sub-G1 phase. LNCaP and C4-2 cells were treated with HPD or control for 24 h. Subsequently, cells were conducted by flow cytometry analysis.

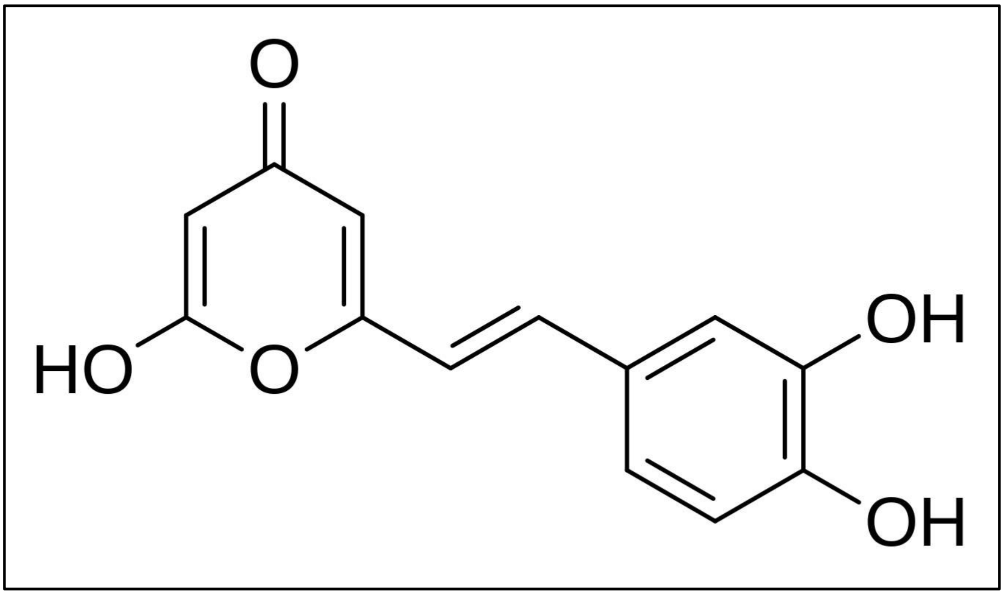

Figure S3. The chemical structure of HPD.
